# Low Prevalence of Brucella agglutinins in Blood Donors in Central Province of Iran

**Published:** 2013-03

**Authors:** Masoomeh Sofian, Mehrnoosh Sheikholeslami, Fatemeh-Alsadat Mahdaviani, Arezoo Aghakhani, Mohammad Banifazl, Ali Eslamifar, Hossein Sarmadian, Ghorban Deiri, Amitis Ramezani

**Affiliations:** 1 Tuberculosis and Pediatric Infectious Research Center, Arak University of Medical Sciences, Arak, Iran; 2 Arak University of Medical Sciences, Arak, Iran; 3 Blood Transfusion Organization Research Center, Advanced Institute for Research and Education in Transfusion Medicine, Tehran, Iran; 4 Clinical Research Dept., Pasteur Institute of Iran, Tehran, Iran; 5 Iranian Society for Support of Patients with Infectious Disease, Tehran, Iran

**Keywords:** *Brucella* agglutinins, blood donors, Brucellosis

## Abstract

**Background and Objective:**

Brucellosis is a zoonotic disease of worldwide distribution and has great economic importance. Despite its control in many countries, it remains endemic in Iran. Brucellosis was investigated in many high risk occupational groups; however, few studies on the prevalence of brucellosis among blood donors are available. To determine the seroprevalence of brucellosis antibodies in blood donors, a serological study was carried out in central province of Iran.

**Materials and Methods:**

A total of 897 healthy blood donors with mean age 37.23± 10.9 years were enrolled in the study. Laboratory tests including Standard Tube Agglutination Test (STA) and 2-mercaptoethanol (2ME) agglutination were checked in all samples. STA dilution ≥1:80, and in the presence of 2-mercaptoethanol (2ME) agglutination ≥ 20 was considered positive.

**Results:**

Out of 897 cases, 11.9% were inhabitants of rural areas. 41.5% had history of consumption of unpasteurized dairy products and 9.3% had history of contact with domestic animals. A very low level of *Brucella* agglutinins was present in 3(0.33%) of the samples and only one sample (0.11%) was found to be truly positive for *Brucella* agglutinins. 2ME was negative in all samples. None of these 4 subjects showed signs and symptoms of brucellosis in 6 months follow-up.

**Conclusion:**

On the basis of our data, brucellosis has no epidemiological and clinical importance in our blood donors; therefore, it is not recommended to perform screening tests such as, STA and 2ME to identify brucellosis antibodies in the sera of blood donors.

## INTRODUCTION

Brucellosis is a zoonotic disease that may have a significant veterinarian, public health and economic impact (1, 2). It can be transmitted to humans through close contact with infected animals or animal products and through the consumptions of unpasteurised milk and dairy products (3). In addition, it is an occupational hazard to persons involved in certain professions such as farming, ranchers, veterinarians and slaughterhouse workers (2, 4).

In humans, brucellosis is an acute febrile illness and behaves as a systemic infection with a very variable clinical spectrum. The pathogen can involve any organ such as the cardiovascular, musculo-skeletal and central nervous systems with sometimes serious complications, and symptoms can vary depending on the site of infection (1, 2, 5).

Brucellosis is endemic in certain parts of Iran. Recent clinical studies have shown that brucellosis is still a common health problem in Iran and sometimes causes severe clinical illness with complications (2, 6, 7). The prevalence of brucellosis in Iran has been reported to be 0.5% to 10.9% in different provinces. It's highly endemic in certain parts of Iran such as central province of Arak (Arak city) with five-year incidence of about 40.5-48/100,000 people (8).

Brucellosis was investigated in many high risk occupational groups; however, few studies on the prevalence of brucellosis among blood donors are available. Determination of the seroprevalence of brucellosis in high risk and low risk groups are very important for understanding of the nature of the disease and eradication of brucellosis. This study was carried out to investigate the background prevalence of Brucella agglutinins in blood donor population in central province of Iran as an endemic area for brucellosis.

## PATIENTS AND METHODS

A total of 897 blood donors attending the Markazi blood transfusion organization were investigated for Brucella agglutinin from April to May 2012. Informed consent was obtained from all cases. The project was approved by Arak University of Medical Sciences ethical committee.

Laboratory tests including Standard Tube Agglutination Test (STA = Wright) and 2-mercaptoethanol (2ME) agglutination were tested in all samples. The STA test was carried out with *Brucella abortus* plain antigen provided by Pasteur Institute of Iran. Serial dilution of the sera in Phosphate buffer saline (PBS) was performed from 1/10 to 1/1280. To each tube 0.5 ml of 10% *Brucella abortus* was added, and incubated at 37° C for 24 h. All the tubes were compared with antigen control tubes for degree of opacity of the supernatant fluid (9). Any serum with titers of 1/80 or above was considered as a positive result.

The 2ME solution was obtained from Pasteur Institute of Iran. The serum treated with 2ME is tested at the same dilutions as STA. To each tube 0.5 ml of 10% *Brucella abortus* was added, and incubated at 37° C for 24 h. The presence of 2ME agglutination ≥20 was considered positive.

All positive samples were following up for 6 months for brucellosis signs and symptoms.

A clinical diagnosis of brucellosis was made on the basis of the symptoms, compatible clinical findings, STA test dilution ≥1:80 and in presence of 2ME agglutination ≥20.

### Statistical analysis

The SPSS 16 Package program for statistical analysis (Chicago, IL, USA) was used. Data are presented as mean ± SD or, when indicated, as an absolute number and percentage.

## RESULTS

Out of 897 cases, with mean age 37.23± 10.9 years, 92.1% were male and 7.9% were female. 11.9% of them were habitant in rural area. 41.5% had history of consumption of unpasteurized dairy products and 9.3% had history of contact with domestic animals. Only 4% of cases had occupational risk for brucellosis acquisition. 5% of subjects had infected family members.

A very low level of *Brucella* agglutinin (1:20) was present in 3 (0.33%) samples from healthy subjects and only one sample (0.11%) was found to be truly positive for *Brucella* agglutinin at 1:80. This sample came from a 26 years old male who lives in Khomein city with history of consumption of unpasteurized milk and milk products and without any history of brucellosis infections and occupational exposure to cattle; but he suffered low back pain. 2ME was negative in all samples. Blood cultures were done in 4 cases and showed negative results. None of these 4 subject revealed signs and symptoms of brucellosis in 6 months follow up.

The *Brucella* agglutinin positive case did not receive any brucella medication because 2ME was negative and his low back pain was mechanical and he did not manifest any brucellosis signs and symptoms in 6 months follow up.

## DISCUSSION

In this study, we investigated the prevalence of Brucella agglutinin in blood donor population in central province of Iran and evaluated the epidemiological, clinical, laboratory findings and outcome of serologic positive cases. This survey showed that the prevalence of Brucella agglutinin was negligible in blood donors in this region and most cases had insignificant levels of Brucella agglutinin, most likely due to endemicity of the disease. Only one sample was found to be truly positive for Brucella agglutinin.

Several studies showed that consumption of unpasteurized dairy products especially in endemic areas is a significant risk factor for brucellosis (2, 10, 11). Brucellosis is also an occupational hazard. Slaughterhouse workers and others involved in animal keeping and handling are at higher risk for disease acquisition (2, 11, 12, 13).

Nikokar et al. (11) reported that the seroprevalence of brucellosis among slaughterhouse workers and the people living in rural areas were 9.8% and 5.5% respectively in North of Iran. The seropositivity rate of 7.8% was reported in high risk groups in South of Iran and they indicated that profession is the main risk factor for seropositivity (14). In a study in Bangladesh, brucellosis seroprevalence determined 11.11% in veterinary personnel, 6.45% in dairy workers and 4.67% in animal farmers (15). In another study on slaughterhouse workers (meat sellers, slaughterers, animal keepers…) in Pakistan, seroprevalence of Brucella antibodies reported 21.7% (16). Swai et al reported that the overall Brucella antibodies seroprevalence in abattoir workers of Tanzania was 5.52% (17).

Bhat et al. (18) reported a seroprevalence rate of 8.5% in the general population of Belgaum. In a study by Ajay Kumar et al (19) on the general population and veterinary students, brucellosis prevalence rate of 2.45% and 1.14% were observed respectively.

There is a paucity of literature on Brucella agglutinin in blood donors. Torres-Padilla et al. (20) determined the Brucella seroprevalence in blood donors of Mexico. They showed brucella seroprevalence in 3.6% of cases. They recommended performing screening tests such as Bengal rose (BR), STA and 2ME to identify brucellosis antibodies in the sera of blood donors. In contrast another study by Vaishnavi et al. (21) in blood donor population of India showed a very low level of Brucella agglutinins in 16.8% of the samples and only one sample (0.36%) was positive for Brucella agglutinins at 160 IU/mL.

Few studies were carried out on Brucella agglutinins prevalence in Iranian blood donors. In a survey conducted by Ghilian et al. (22) in blood donors of Yazd, 6.3% of participants had a STA titer of 1:80 but 2-ME test was positive only in 0.6% of cases.

In contrast, Rabbani Khorasgani et al. screened blood donors of Boushehr province (south of Iran) for serological evidence of brucellosis. Brucella agglutinins found in 0.057% of sera samples and 2ME test was positive only in one sample (from 10500 blood donors) with low titer (1/20) without any symptoms of active infection (23).

The young male in our study with positive Brucella agglutinin lives in the city and have not provided any past history of brucellosis, cattle contact and high risk occupation for acquisition of the disease; but this seropositivity may be due to consumption of unpasteurized milk and milk products or exposure to an agricultural background.

In conclusion on the basis of our data, brucellosis has no epidemiological and clinical importance in our blood donors; therefore it is not recommended to perform screening tests such as STA and 2ME to identify brucellosis antibodies in the sera of blood donors.
